# TLR7/TLR8 activation and susceptibility genes synergize to breach gut barrier in a mouse model of lupus

**DOI:** 10.3389/fimmu.2023.1187145

**Published:** 2023-07-06

**Authors:** Longhuan Ma, Morgan Terrell, Josephine Brown, Abigail Castellanos Garcia, Ahmed Elshikha, Laurence Morel

**Affiliations:** ^1^ Department of Microbiology, Immunology, and Molecular Genetics, University of Texas Health San Antonio, San Antonio, TX, United States; ^2^ Department of Pathology, Immunology, and Laboratory Medicine, University of Florida, Gainesville, FL, United States

**Keywords:** lupus, TLR7/8 activation, gut barrier, lymphocytes, NKp46^+^ cells

## Abstract

**Background:**

Mounting evidence suggests that increased gut permeability, or leaky gut, and the resulting translocation of pathobionts or their metabolites contributes to the pathogenesis of Systemic Lupus Erythematosus. However, the mechanisms underlying the induction of gut leakage remain unclear. In this study, we examined the effect of a treatment with a TLR7/8 agonist in the B6.*Sle1.Sle2.Sle3* triple congenic (TC) mouse, a spontaneous mouse model of lupus without gut leakage.

**Materials and methods:**

Lupus-prone mice (TC), TC.*Rag1^-/-^* mice that lack B and T cells, and congenic B6 healthy controls were treated with R848. Gut barrier integrity was assessed by measuring FITC-dextran in the serum following oral gavage. Claudin-1 and PECAM1 expression as well as the extent of CD45^+^ immune cells, B220^+^ B cells, CD3^+^ T cells and CD11b^+^ myeloid cells were measured in the ileum by immunofluorescence. NKp46^+^ cells were measured in the ileum and colon by immunofluorescence. Immune cells in the ileum were also analyzed by flow cytometry.

**Results:**

R848 decreased gut barrier integrity in TC but not in congenic control B6 mice. Immunofluorescence staining of the ileum showed a reduced expression of the tight junction protein Claudin-1, endothelial cell tight junction PECAM1, as well as an increased infiltration of immune cells, including B cells and CD11b^+^ cells, in R848-treated TC as compared to untreated control mice. However, NKp46^+^ cells which play critical role in maintaining gut barrier integrity, had a lower frequency in treated TC mice. Flow cytometry showed an increased frequency of plasma cells, dendritic cells and macrophages along with a decreased frequency of NK cells in R848 treated TC mice lamina propria. In addition, we showed that the R848 treatment did not induce gut leakage in TC.*Rag1^-/-^* mice that lack mature T and B cells.

**Conclusions:**

These results demonstrate that TLR7/8 activation induces a leaky gut in lupus-prone mice, which is mediated by adaptive immune responses. TLR7/8 activation is however not sufficient to breach gut barrier integrity in non-autoimmune mice.

## 1 Introduction

Systemic lupus erythematosus (SLE) is a chronic autoimmune disease characterized by the production of autoantibodies and tissue damage induced by immune complex deposition (1). Genetic as well as environmental factors contribute to the initiation and progression of disease. Loss of gut barrier integrity has been reported in SLE patients as well as in several mouse models of the disease, and it has been proposed that microbial translocation out of the gut contributes to lupus pathogenesis (2). Bacterial components have been detected in the blood of lupus patients (3, 4). In addition, *Enterococcus gallinarium and Lactobacillus reuteri* are pathobionts that have been detected in the internal organs of two mouse models lupus (5, 6). *E. gallinarum* was also detected in the liver of patients with autoimmune hepatitis, and human hepatocytes cultured with *E. gallinarum* produced multiple autoimmune-promoting factors, including type I IFN (5). Since whole bacteria or their components are strong proinflammatory mediators, their presence in internal organs and the circulation may trigger inflammation, resulting in the loss of host immune tolerance or the amplification of an existing autoimmune activation. The mechanisms responsible for gut leakage in SLE are not well-understood, although it is recognized that the balance of anti- and pro-inflammatory factors is critical to maintain gut function and integrity (7). It has been proposed that specific bacteria enriched in the gut of SLE patients may directly contribute to the loss of barrier integrity. In support of this hypothesis, gut leakage was induced in gnotobiotic non-autoimmune mice by the colonization with a strain of *Ruminococcus gnavus* that blooms in patients with lupus nephritis (8). The inflammatory milieu that develops in the gut of SLE patients may also contribute to the loss of barrier integrity.

Toll like receptors (TLRs) play a crucial role in the recognition of pathogen-associated molecular patterns (PAMPs) and the activation of the immune response. It is well known that interactions between TLRs and gut microbiota help to maintain gut homeostasis. Overstimulation of TLRs may disturb a balanced composition of the gut microbiome (9), which may in turn promote gut permeability. TLR activation has also been implicated both in the loss and the maintenance of the gut integrity in mice. TLR4 activation by LPS increased the colonic paracellular permeability while activating TLR2 alleviated gut leakage (10). In addition, intracolonic activation of TLR7 increased gut permeability without altering the expression of tight junction proteins, suggesting that other mechanisms were involved (11).

TLR7 overactivation is tightly linked to lupus pathogenesis, with a gain of function mutation resulting in a monogenic pediatric SLE (12) and *Tlr7* duplication in the *Yaa* locus or transgenic expression resulting in lupus phenotypes in mice (13). Moreover, a topical treatment with Resiquimod (R848), a TLR7/8 agonist, induces lupus-like manifestations in non-autoimmune mice (14). The two lupus-prone mouse strains in which the spontaneous translocation of pathobionts has been documented either carry the *Yaa* locus (5) or a *Tlr7* transgene (6). However, whether TLR7 activation played a role in this pathogenic trigger was not specifically addressed. We have shown that the B6.*Sle1.Sle2.Sle3* triple congenic (TC) mouse model of lupus presents a gut bacterial dysbiosis that induces autoimmune activation upon transfer into non-autoimmune congenic control C57BL/6 (B6) mice (15). Gut permeability is not impaired in this model, suggesting that other mechanisms, including an altered microbial tryptophan metabolism, are responsible for the dysbiotic autoimmune activation (15, 16). We have shown that topical R848 greatly accelerated disease in TC mice, including the development of cardiovascular pathology that did not develop in R848-treated B6 mice (17). This indicated that a lupus-prone genetic background potentiates the inflammatory consequences of the activation of the TLR7/8 pathway. Using this model, we found in this study that activation of the TLR7/8 pathway impaired gut permeability, but only in the presence of a lupus-prone genetic background. We also showed that this TLR7/8-induced inflammation of the gut did not occur in Rag1-deficient TC mice. TLR7/8 activation remodeled the distribution of immune cells in the gut epithelium with an increase of dendritic cells (DCs), macrophages and plasma cells, and a loss of natural killer (NK) cells. These results indicate that the activation of the TLR7/8 pathway that frequently occurs in lupus pathogenesis is likely to contribute to the loss of gut barrier integrity, and that it depends on the presence of lymphocytes and the expression of lupus-susceptibility genes.

## 2 Materials and methods

### 2.1 Mice and treatment

C57Bl/6J (B6) were purchased from The Jackson Laboratories. B6.*Sle1.Sle2.Sle3* (TC) and TC.*Rag*1^-/-^ mice have been described previously (18, 19). All mice were bred and maintained at the University of Florida in specific pathogen-free conditions. Both males and females were used with gender and age-matched controls for each experiment. Mice from each strain used in this study were strictly maintained with littermates or mice from the same strain within the same group (i.e. less than 4 weeks apart). This housing policy was to avoid transfer of autoimmune activation or attenuation that occurs between B6 and TC mice that share their microbiome (15). rTC mice between 6 and 12 weeks of age, before they produce anti-dsDNA IgG (referred to as “pre-autoimmune”), along with B6 mice were treated with 100 μg resiquimod (R848; Tocris) in 100 μl acetone (Thermo Fisher Scientific) by topical application to the right ear three times a week for 2 weeks. Control mice were left untreated. The tissues were harvested 2 days after the last treatment. Before euthanasia, the gut permeability was evaluated by gavaging mice fasted for 4 h with 5 mg FITC-dextran 4000 (Sigma-Aldrich) in 200 μl 1x PBS, which was quantified in the serum 2 h later by flow cytometry. This study was carried out in accordance with the guidelines from the Guide for the Care and Use of Laboratory Animals of the Animal Welfare Act and the National Institutes of Health. All animal protocols were approved by the Institutional Animal Care and Use Committee of the University of Florida.

### 2.2 Intestinal tissue harvest and immunostaining

Ileum and colon were prepared as “Swiss roll” before being embed in OCT medium (Fisher Scientific) and snap-frozen at − 80 °C. 7 µm thick sections mounted on histology slides were put at room temperature for 20 min before being placed into PBS to dissolve the OCT. The sections were fixed with cold acetone for 10 min., washed 3 times wash, and blocked with 10% normal rat serum (Equitech-Bio) in PBS for 30 min. The sections were then stained with antibodies to CD45 (1:25 dilution; Biolegend 30-F11), B220 (1:50; Southern Biotech RA3-6B2), CD3 (1:50; eBioscience 145-2C11), Claudin-1(1:100 dilution; Invitrogen MH25), NKp46 (1:100 dilution; R&D Systems MAB22252), and PECAM1 (1:50 dilution; BD Biosciences MEC13.3). Fluorescence intensity was analyzed using ImageJ.

### 2.3 Flow cytometry

Single-cell suspensions were isolated from the gut by using the lamina propria dissociation kit with gentleMACS tissue dissociator (Miltenyi Biotech). Cells were stained in 2.5% FBS and 0.05% sodium azide in PBS. Fluorochrome-conjugated antibodies were as follows: B220 (RA3-6B2), CD11b (M1/70), CD11c (HL3), CD3e (145-2C11), CD95 (Jo2), CD19 (eBio1D3), CD8a (53-6.7), Ly6G (1A8) and Siglec-F (E50-2440) were purchased from BD Biosciences. CD4 (RM4-5), CD138 (281–2), MHCII (M5/114.15.2), NK.1.1 (PK 136), CD49b (HMa2), IL-10 (JES5-16E3), IL-17 (TC11-18h10.1), F4/80 (BM8) and IFN-γ (XMG1.2) were purchased from BioLegend. Foxp3 (FJK-16S), GL-7 (GL-7), CD45 (104) and PDCA-1 (eBio927) were purchased from eBioscience. Dead cells were excluded with fixable viability dye (eFluor780; Thermo Fisher Scientific). Intracellular staining was performed with a fixation/permeabilization kit (eBioscience). For cytokine detection, splenocytes were stimulated with the Leukocyte Activation Cocktail (BD Biosciences) at 37 ^0^C for 4 h. All samples were acquired on an LSRFortessa flow cytometer (BD Biosciences) and analyzed with FlowJo software (Tree Star). Gating strategies are shown in **Figures S1**, **S2**. Cell counts for each cell population were calculated based on the splenocyte cell counts measured with a Cellometer Auto 2000 (Nexcelom) and the frequencies of these populations based on the gates shown in Figures S1, **S2**.

### 2.4 Detection of IL-6 by ELISA

Serum IL-6 was quantitated with the IL-6 ELISA kit (BD Biosciences) according to manufacturer’s instructions. Serum samples were diluted 1:50 and assayed in duplicate. The absorbance was detected with the Promega GloMax^®^ Explorer microplate reader at 450 nm.

### 2.5 Statistics

Statistical analyses were performed with the Graphpad Prism 9.0 software. Differences between groups were evaluated by one-way ANOVA with correction for multiple tests, or unpaired t tests, as indicated in the text. Unless specified, all tests are two-tailed. Results were expressed as means ± standard deviation. The levels of statistical significance were set at *: *P* < 0.05, **: *P* < 0.01, ***: *P* < 0.001 and ****: *P* < 0.0001.

## 3 Results

### 3.1 Topical TLR7/8 activation enhanced gut permeability in lupus-prone TC mice

**Figure 1:**
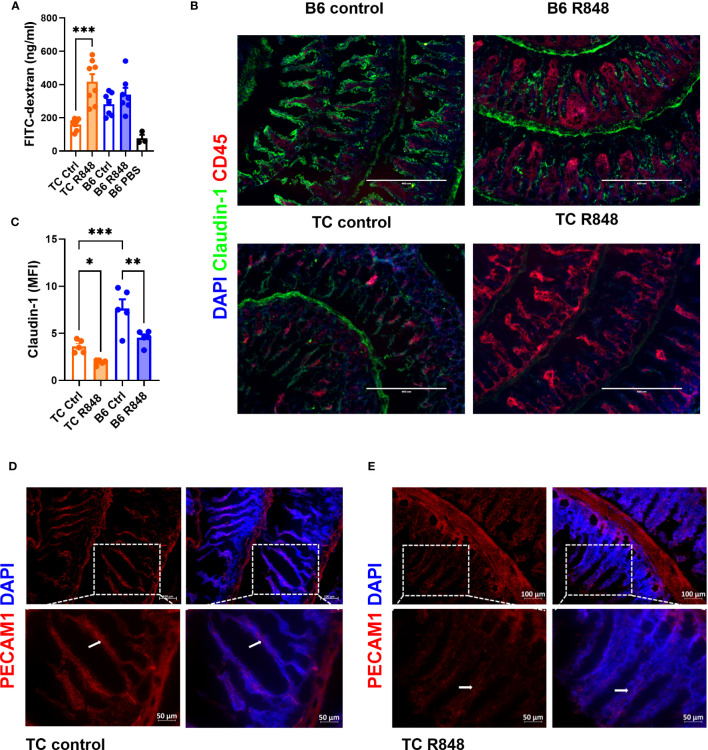
TLR7/8 activation enhanced gut permeability in lupus-prone TC mice. **(A)** FITC-dextran concentration in the serum of TC or B6 mice with or without R848 treatment. The results were from two cohorts of mice. **(B)** Representative immunostainings of tight junction protein Claudin-1 and immune cell marker CD45 in the ileum of TC and B6 mice with or without R848 treatment. **(C)** Quantification of Claudin-1 in the ileum of the TC or B6 mice with or without R848 treatment. **(D, E)** Representative immunostainings of endothelial tight junction protein PECAM1 (red) in the ileum of control **(D)** and treated **(E)** TC mice. The dashed boxes indicate the location of the higher magnification images shown below. Arrows point to PECAM1 labeling. N = 3 - 5. Mean + SEM compared with 1-way ANOVA with multiple-comparison tests. *: P < 0.05; **: P < 0.01; ***: P < 0.001.

To assess the effect of TLR7/8 activation on gut integrity, we treated pre-autoimmune TC and age-matched control B6 mice with R848 applicated to the ear for 2 weeks as previously described (17). Untreated mice were used as controls. At the end of the treatment, the amount of FITC-dextran in the serum following oral gavage was measured to assess gut permeability. R848 increased the level of FITC-dextran in the serum of TC mice but not in B6 mice (**Figure 1A**). Claudin-1, one of the tight junction proteins that maintains the intestinal barrier, was quantified in the ileum by immunofluorescence. Claudin-1 levels were lower in untreated TC than untreated B6 mice, and they were further reduced by the R848 treatment (**Figures 1B, C**). Similarly, the expression of PECAM1, a tight junction protein between endothelial cells, was also decreased by R848 treatment in the blood and/or lymphatic vessels in the intestinal lamina propria (**Figures 1D, E**). The treatment induced a heavy CD45^+^ immune cell infiltration in the gut, albeit without statistical difference between the strains. Taken together, the combination of TLR7/TLR8 activation and lupus susceptibility genes expressed in TC mice functionally breached the gut barrier and decreased the expression of tight junction proteins.

### 3.2 Both innate and adaptive immune cells were recruited by TLR7/8 activation to the ileum of TC mice

**Figure 2:**
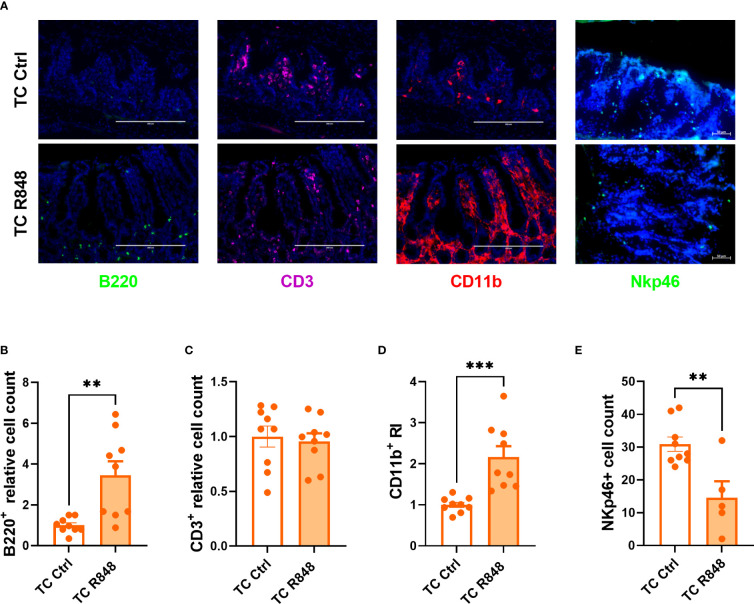
Immune cell recruitment by TLR7/8 activation to the gut of TC mice. Representative images **(A)** and quantitation **(B–E)** of immunofluorescence staining of B220^+^, CD3^+^, CD11b^+^ **(B–D)** and NKp46^+^ cells in the ileum and colon **(E)** of TC mice with or without R848 treatment. The relative cell counts in panel **(B, C)** were calculated relative to the mean of control groups. N = 3. Mean + SEM compared with t tests. **: P < 0.01; ***: P < 0.001.

We used immunofluorescence to characterize the massive immune cell infiltration induced by the R848 treatment in the ileum of TC mice (**Figure 2A**). A significantly higher number of B220^+^ B cells was detected in the lamina propria of treated mice, with notably most of them located towards the bottom of the villi (**Figure 2B**). CD3^+^ T cells were distributed evenly across the lamina propria and the R848 treatment did not change their number (**Figure 2C**). The number of CD11b^+^ cells aggregated in the lamina propria was higher in R848-treated than control TC mice (**Figure 2D**). We also investigated the NKp46^+^ cell population, which includes a subset of NK cells and innate lymphoid cells (ILC), and plays a critical role in the maintenance of gut barrier integrity (20, 21). A high number of NKp46^+^ cells were found in the colon of TC mice that was decreased by the R848 treatment (**Figure 2E**). NKp46^+^ cells were not detected by histology in the ileum of TC mice (data not shown). Overall, TLR7/8 activation mediated the infiltration of B cells and innate immune cells in the gut of TC mice but decreased NKp46^+^ cells.

**Figure 3:**
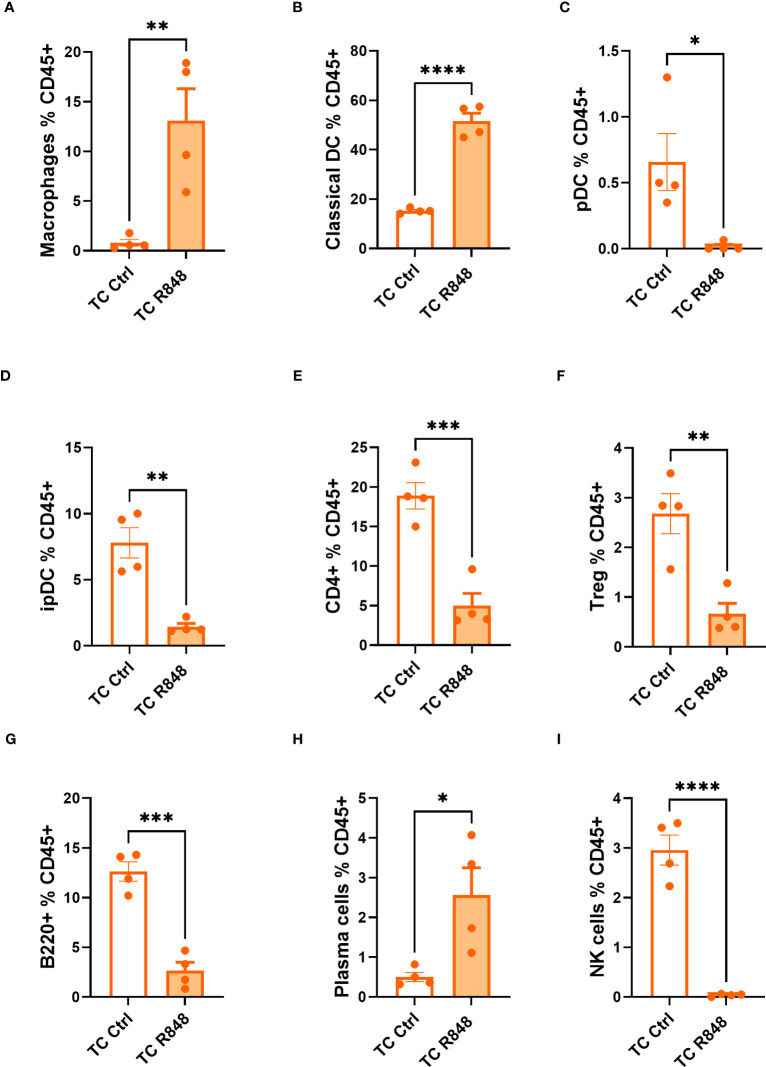
Immunophenotyping of the ileum lamina propria comparing control (Ctrl) and treated (R848) TC mice. Frequency of macrophages **(A)**, cDCs **(B)**, pDCs **(C)**, ipDCs **(D)**, CD4^+^ T cells **(E)** and Treg cells **(F)** out of CD45^+^ cells. The frequency of B cells **(G)**, plasma cells **(H)**, and NK cells out of CD45^+^ cells **(I)**. N = 4. Mean + SEM compared with t tests. *: P < 0.05; **: P < 0.01; ***: P < 0.001; ****: P < 0.0001.

The cells in the lamina propria mice were further compared between treated and control TC mice by flow cytometry. A higher frequency of macrophages and conventional dendritic cells (DCs) was found in treated TC mice (**Figures 3A, B**, **Figure S3A, B**). In comparison, the treatment reduced the frequency of both plasmacytoid dendritic cells (pDCs) and inflammatory pDCs (ipDCs) gated as defined in (22, 23) and shown in **Figure S2** (**Figures 3C, D**, **Figures S3C, D**). Since pDCs are the main producers of type I IFN in response to TLR7 activation (24), it is possible that R848 induced their recruitment and migration outside the gut. Similarly, the frequency of total CD4^+^ T cells and regulatory T (Treg) cells among CD45^+^ cells was reduced by the R848 treatment (**Figures 3E, F**, **Figures S3D, E**). The R848 treatment did not affect however the frequency of Treg cells among CD4^+^ T cells in TC mice (**Figure S4A**). In addition, the frequency of CD8^+^ T cells followed the same trend as total CD4^+^ T cells (**Figure S4B**). There was a trend of reduced numbers of CD4^+^ and CD8^+^ T cells in the lamina propria, but the differences were not significant (**Table 1**). The R848 treatment also decreased the frequency of B cells while significantly increasing the frequency of plasma cells (**Figures 3G, H**, **Figures S3F, G**), consistent with TLR7/8 activation. Finally, the R848 treatment virtually eliminated NK cells from the ileum of TC mice (**Figure 3I**, **Figure S3H**). The frequency of neutrophils and eosinophils in the ileum of TC mice were not affected by the R848 treatment (**Figures S4C-F**). Similar results were obtained with cell numbers (**Table 1**). These observations show a complex pattern of modifications of immune cells in the gut of TC mice by TLR7/8 activation.

**Table 1:** Immune cells count in the lamina propria of treated and control TC mice.

	TC Ctrl	TC R848
cDCs	324.00 ± 56.65	999.30 ± 262.8^*^
pDCs	13.75 ± 4.48	0.25 ± 0.25^*^
ipDCs	180.80 ± 54.87	30.50 ± 12.55^*^
CD4^+^ T cells	301.00 ± 63.02	111.00 ± 68.38
CD8^+^ T cells	110.80 ± 28.46	52.25 ± 37.18
Treg cells	43.75 ± 10.72	14.75 ± 9.11
B220^+^ cells	287.50 ± 68.54	82.50 ± 31.17^*^
Plasma cells	11.50 ± 4.63	72.25 ± 16.54^*^
NK cells	69.00 ± 18.61	1.25 ± 0.63^*^
Macrophages	18.25 ± 9.707	331.00 ± 129.80
Neutrophils	9.75 ± 1.44	5.75 ± 1.80
Eosinophils	3.50 ± 1.32	18.00 ± 6.65

N = 4. Mean + SEM compared with t tests. *: P < 0.05.

### 3.3 Lymphocytes are required for R848-mediated gut permeability in TC mice

**Figure 4:**
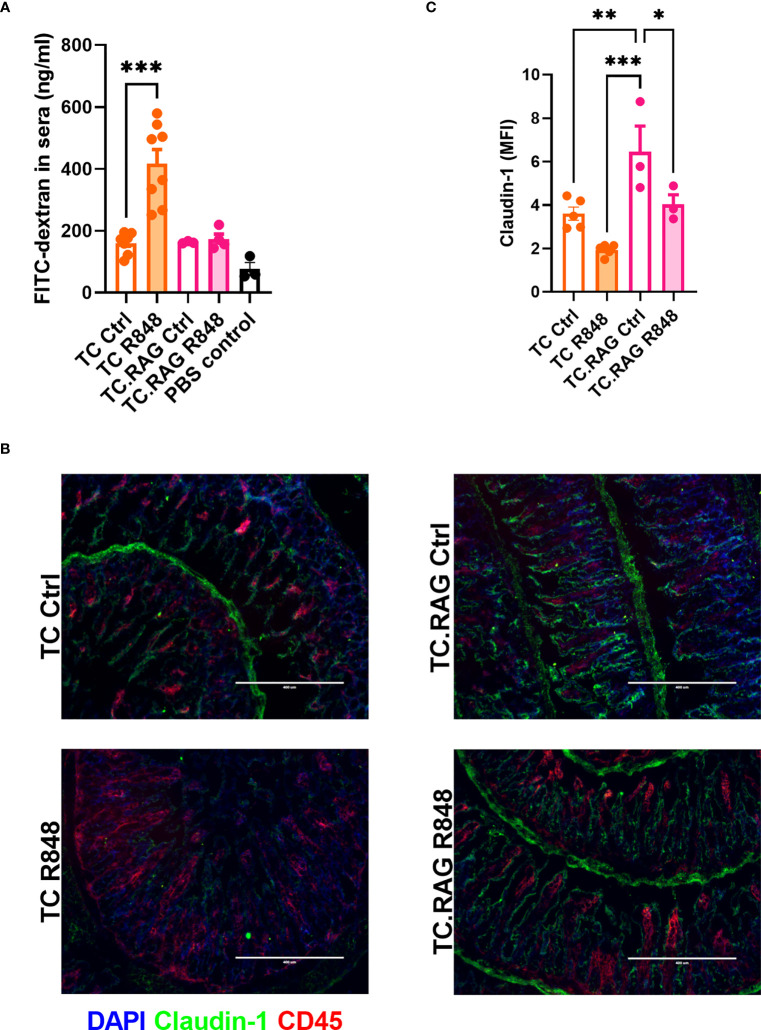
Adaptive immunity is required in R848-mediated gut permeability in TC mice. **(A)** FITC-dextran concentration in the serum of TC or TC. Rag1^-/-^ mice with or without R848 treatment. **(B)** Representative immunostainings of tight junction protein Claudin-1 and immune cell marker CD45 in the ileum of TC and TC. Rag1^-/-^ mice with or without R848 treatment. **(C)** Quantification of Claudin-1 protein. N = 3 - 5. Mean + SEM compared with t tests. *: P < 0.05; **: P < 0.01; ***: P < 0.001.

The inflammatory cascade induced by either immune complexes or the activation of innate cells causes tissue injury that can decrease gut integrity (25). To dissect the relative roles of innate and adaptive immunity in R848-mediated gut leakage in TC mice, we evaluated B and T-cell deficient TC.*Rag1^-/-^* mice. The R848 treatment did not increase the amount of FITC-dextran leaked in the serum of TC.*Rag1^-/-^* mice as it did for TC mice (**Figure 4A**), suggesting that B cells, T cells, or both were involved in the TLR7/8-mediated gut leakage in TC mice. We also evaluated the level of serum IL-6, which can be produced by B and T cells, and is elevated in lupus (26). High levels of IL-6 have been associated with decreased gut barrier integrity, including in a model of lupus (27, 28). Il-6 levels were similar between groups (**Figure S5**), excluding a casual role of IL-6 on gut leakage in this model. Immunofluorescence staining showed that the R848 treatment failed to recruit CD45^+^ immune cells to the gut of TC. Rag1^-/-^ mice as it did in TC mice. Moreover, the expression of Claudin-1 was significantly higher in TC. *Rag1^-/-^* than TC ileums, although it was decreased by the R848 treatment in both strains (**Figures 4B, C**). The results suggest that adaptive immunity is required in TLR7/8 signaling pathway activation mediated gut leakage in TC mice.

## 4 Discussion

TLR7/8 activation impaired gut integrity in lupus-prone TC mice but not in congenic controls based on the FITC-dextran assay, and it lowered the expression of Claudin-1 between gut epithelial cells and PECAM1 between blood/lymphatic endothelial cells. These results demonstrated that TLR7/8 activation compromises gut barrier integrity in mice that express lupus susceptibility genes, but it is not sufficient on a non-autoimmune genetic background. Since a control group of mice treated with acetone alone was not included in the study, these results should however be interpreted with caution. Mechanistically, the lupus genetic background could license the breach of gut barrier integrity in response to TLR7/8 activation through the gut dysbiosis that is present in TC mice, and that can transfer autoimmune activation in non-autoimmune mice (15). It is conceivable that the TC gut microbiota was further modified by TLR7/8 activation to expand bacteria that breach the epithelial barrier. NKp46^+^ cells, which are critical in maintaining barrier integrity depend on the microbiota to differentiate (29). A TRL7/8 induced alteration of the TC microbiota could result in the observed decreased NKp46^+^ cell frequency, which in turn, may fail to maintain barrier integrity. Testing these hypotheses will require at minimum to assess gut permeability in control mice after fecal microbiome transfers from R848-treated TC mice.

The lupus genetic background could also license the breach of gut barrier integrity in response to TLR7/8 activation through the immune system. TC mice present a heightened immune activation at baseline (18) and in response to TLR7/8 activation (17). Our group has reported an enhanced expression of type 1 interferon stimulated genes in the heart of R848-treated TC mice (17). Considering the dual roles of type 1 interferon on gut inflammation and injury (30), the activation of this pathway in TC mice may partially explain the increased gut leakage in this model. TLR7 and TLR8 are widely expressed in gut myeloid cells, lymphocytes, and dendritic cells, as well as intestinal epithelial cells (IEC), with the highest expression levels in the latter two populations (31). The finding that the R848 treatment does not induce gut leakage in TC.*Rag1^-/-^* mice strongly suggests that the activation of B cells and/or T cells by TLR7/8 pathway is directly or indirectly required for the induction of gut leakage. By histology, we found that the R848 treatment increased the numbers of B cells and CD11b^+^ myeloid cells, but not CD3^+^ T cells in the ileum of TC mice. Flow cytometry showed an increased frequency of plasma cells but a decreased frequency of B cells. Previous studies have demonstrated that R848 stimulation could initiate B cell proliferation, upregulation of costimulatory molecule expression and antibody production (32). Our observations suggest that the R848-treatment triggers a robust differentiation into plasma cells in the gut of TC mice. Antibodies or autoantibodies may regulate gut homeostasis through their interactions with bacteria (33). Secretory Immunoglobulin A (SIgA) derived from intestinal plasma cells binds to pathogenic microbes, preventing their expansion and keeping the gut homeostasis (34). However, antibodies produced by plasma cells in the lamina propria may form immune complexes that may induce tissue damage and increase gut permeability. In support of this hypothesis, the number of IgA-producing plasma cells was positively correlated with the gut permeability in human duodenal biopsies (35).

Flow cytometry also showed a global decrease in T cell frequency in the ileum of R848-treated TC mice, including in Treg cell frequency relative to the total immune infiltrate. The discrepancy showing a similar number of T cells by immunofluorescence and a decreased by flow cytometry deserves to be examined further in futures studies. Flow cytometry is likely to be more reliable as more quantitative. Treg cells have been proposed to be central to the interplay between the host and microbial milieu. These cells are involved in promoting gut barrier integrity and a balanced interaction with gut microbiota–derived short-chain fatty acids (SCFAs) (36). The decreased number of Treg cells in R848-treated TC mice may contribute to an enhanced gut inflammation, leading to deleterious gut integrity, although their frequency relative to effector T cells was not changed. However, Treg cells in the gut are heterogenous in their origin, function and interactions with the microbiome (37). A more detailed analysis will be therefore necessary to assess whether a loss of Treg cells in R848-treated TC mice plays a role in their gut leakage. Several T cell-derived cytokines contribute to the maintenance of gut integrity, including IL-10, IL-17 and IL-22 (38–40). The decreased frequency of TC CD4^+^ T cells in the gut by TLR7/8 activation may skew the cytokine environment. Ultimately, this altered cytokine milieu may affect gut integrity directly or modify other cell populations through downstream signaling pathway and eventually make an impact on gut barrier function.

The gut myeloid populations of TC mice showed a heterogeneous response to R848 treatment, which included an increased frequency of macrophages and cDCs. R848 activates the NF-κB pathway (41) as well as the production of IL-1β and IL-18 (42), and promotes an M1 phenotype (43), all of which are likely to amplify inflammation or cause tissue damage (44). cDCs work as professional antigen-presenting cells for T cell priming. Interactions of resting immature DCs with TLR ligands leads to a cascade of pro-inflammatory cytokines and skewing of T cell responses. Interactions between cDCs and bacteria may alter cDC activation and indirectly shape CD4^+^ T cell differentiation and effector functions. Specific components of the gut microbiota isolated from patients with celiac disease altered DC maturation and their interactions with epithelial cells, leading to gut permeability (45). In a model of gut inflammation, DC activation and aberrant distribution induced by acute antigen uptake has been correlated to an enhanced gut permeability (46). In the small intestine, lamina propria dendritic cells (LPDCs) induce Treg cell differentiation. Over-reactivated LPDCs may lose their tolerogenic function, leading to a decrease of Treg cell number (47). In the present study, an enhanced DC activity may explain the reduced number of Treg cells, both of which may contribute to an increased inflammatory state. At same time, a decreased pDC frequency was observed in the gut of R848-treated TC mice. We have shown that R848 expanded the pDC population in the spleen of TC mice to a greater extent than in B6 spleens (17). TLR7 activation suppressed the expression of CCR9, the gut homing receptor, on pDCs (48). This observation may at least partially explain the decreased frequency of pDCs in the gut of R848-treated TC mice.

Finally, we observed that the R848 treatment greatly reduced the NKp46^+^ cell population in the colon and the NK cells in the ileum of TC mice. Cytokines, including IL-22, that are produced by NK cells and ILCs are involved in the regulation of various immune cells maintaining the integrity of the gut mucosa (49). ILC3 cells have been related to the gut integrity maintenance (50). Altered gut permeability was associated to a reduced NK cell number in schizophrenia patients (51). Therefore, the decreased number of NKp46^+^ cells in the gut of R848-treated TC mice may partially explain the increased gut permeability. It should be noted that although the results obtained with TC.*Rag1^-/-^* mice indicate that B and/or T cells play a major role in breaching the gut barrier in response to R848, cDCs, macrophages, and NKp46^+^ cells may play an indirect role by altering the lymphocyte populations. For instance, cytokines produced by T and B cells may modify activation of DCs and macrophages. Which immune cell population individually or collectively contribute to gut permeability needs to be addressed with selective elimination with lineage specific antibodies.

In summary, we showed that the activation of the TLR7/8 pathway, a common feature of lupus pathogenesis, may contribute to impaired gut barrier integrity but only in the presence of lupus genetic susceptibility. It would be of great interest to evaluate whether SLE patients with an activated TLR7/8 pathway, most likely measured as its downstream type I IFN activity, present with an increased incidence of leaky gut. The interpretation of such a study may however be difficult because such patients tend to also present with a higher disease activity and inflammation, which may be a contributing factor to leaky gut independently from TLR7/8.

## Data availability statement

The raw data supporting the conclusions of this article will be made available by the authors, without undue reservation.

## Ethics statement

The animal study was reviewed and approved by the Institutional Animal Care and Use Committee of the University of Florida (IACUC 202009466).

## Author contributions

LMa, MT, JB, AE, and LMo designed the experiments and analyzed results. LMa, MT, JB, and AE conducted experiments. LMa and LMo wrote the manuscript. All authors contributed to the article and approved the submitted version.

## Supplementary material

Supplementary Figure 1 Gating strategies for lamina propria immunophenotyping.

Supplementary Figure 2 Gating strategy for dendritic cell populations.

Supplementary Figure 3 Representative FACS plots for macrophage **(A)**, cDCs**(B)**, pDCs & ipDCs **(C)**, CD4+ & CD8+ **(D)**, Treg **(E)**, B cells **(F)**, plasma cells & plasma blast cells **(G)** and NK cells **(H)**.

Supplementary Figure 4 Frequency of Treg **(A)**, CD8^+^ T cells **(B)**, neutrophils **(C)** and eosinophils **(D)** in the ileum of TC mice with or without R848 treatment and representative FACS plots for neutrophils **(E)** and eosinophils **(F)**. N = 4. Mean + SEM.

Supplementary Figure 5 Serum IL-6 levels in B6, TC or Tc. Rag^-/-^ mice with or without R848 treatment. N = 3 - 4. Mean + SEM.
