# Supplementary material for: TLR7/TLR8 activation and susceptibility genes synergize to breach gut barrier in a mouse model of lupus
Source: Front Immunol. 2023 Jul 6;14:1187145. doi: 10.3389/fimmu.2023.1187145 (PMC10358848; doi:10.3389/fimmu.2023.1187145)
Supplement: Supplementary Figure 1 — Gating strategies for lamina propria immunophenotyping. [file Image_1.pdf]

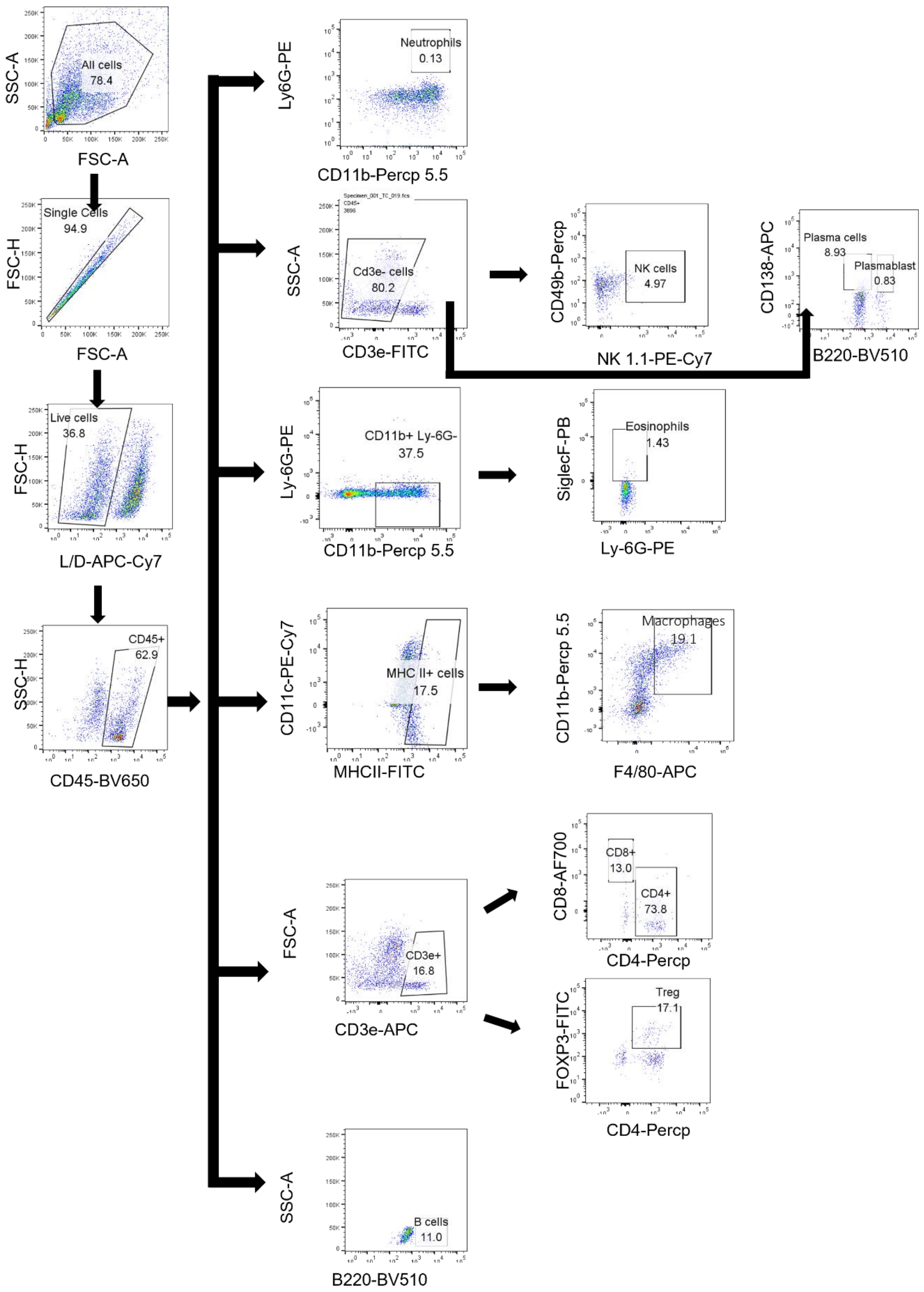

Figure S1. Gating strategies for lamina propria immunophenotyping.

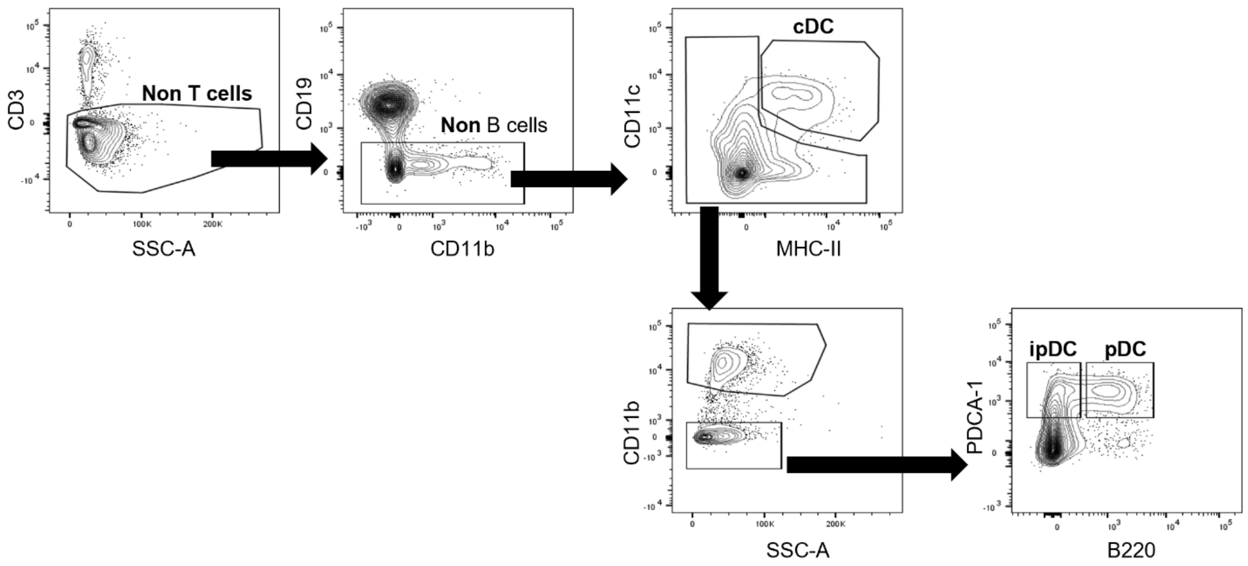

Figure S2. Gating strategy for dendritic cell populations.

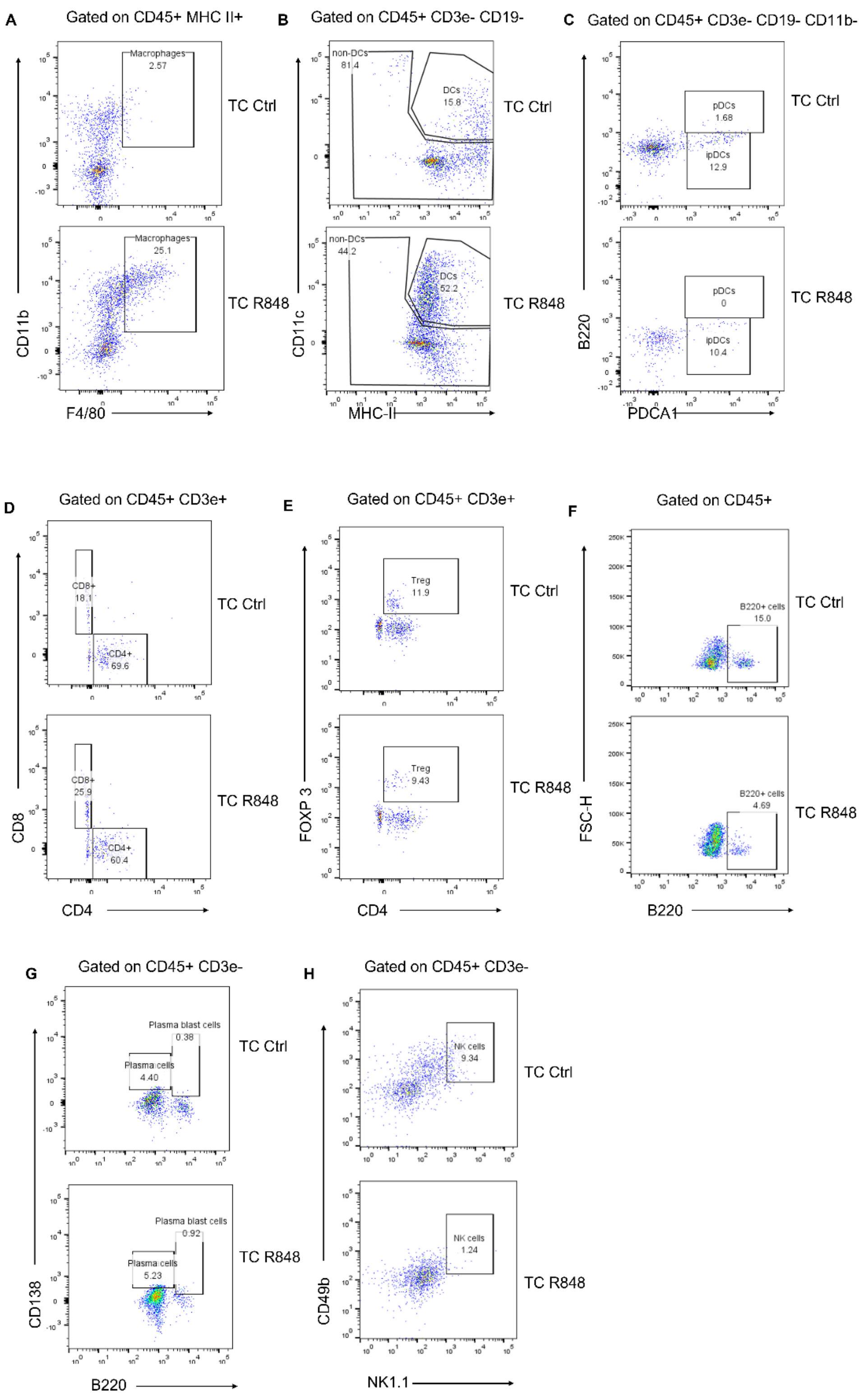

Figure S3. Representative FACS plots for macrophage (A), cDCs(B), pDCs & ipDCs (C), CD4+ & CD8+ (D), Treg (E), B cells (F), plasma cells & plasma blast cells (G) and NK cells (H).

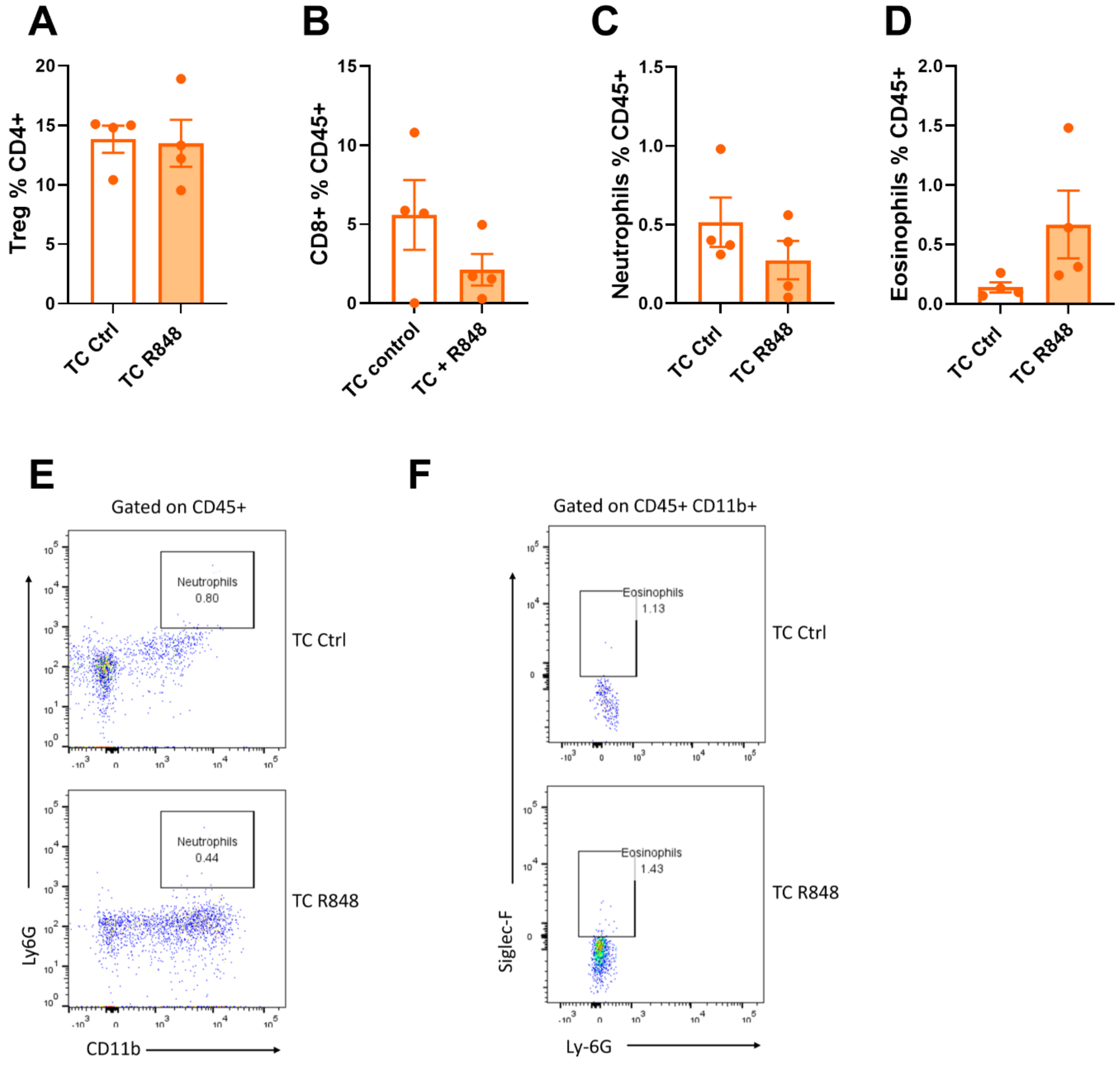

Figure S4. Frequency of Treg (A), CD8⁺ T cells (B), neutrophils (C) and eosinophils (D) in the ileum of TC mice with or without R848 treatment and representative FACS plots for neutrophils (E) and eosinophils (F). N = 4. Mean + SEM.

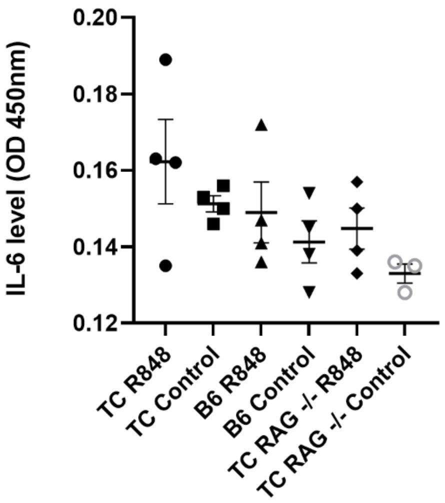

Figure S5. Serum IL-6 levels in B6, TC or Tc. Rag$^{-/-}$ mice with or without R848 treatment. N = 3 - 4. Mean + SEM.
